# Immunological factors influencing HIV acquisition in infants: A narrative review

**DOI:** 10.1097/MD.0000000000043039

**Published:** 2025-06-20

**Authors:** Emmanuel Ifeanyi Obeagu, Isaac Isiko, Getrude Uzoma Obeagu

**Affiliations:** a Department of Biomedical and Laboratory Science, Africa University, Mutare, Zimbabwe; b Department of Community Medicine, School of Public Health, NIMS University, Jaipur, India; c School of Nursing Science, Kampala International University, Ishaka, Uganda.

**Keywords:** HIV, immune response, infants, maternal antibodies, mucosal immunity

## Abstract

Human immunodeficiency virus (HIV) acquisition in infants is a critical public health concern, particularly in regions with high maternal HIV prevalence. The susceptibility of infants to HIV is influenced by a complex interplay of immunological factors, including maternal antibodies, innate immune responses, and the development of adaptive immunity. This review aims to elucidate the various immunological components that contribute to the risk of HIV transmission in infants and highlights the importance of understanding these factors for developing effective prevention strategies. Maternal antibodies provide passive immunity to infants, protecting them from infections during the early months of life. However, the presence of these antibodies can also modulate the infant’s immune responses, potentially affecting their susceptibility to HIV. The innate immune system, characterized by the activity of natural killer cells and other immune cells, serves as the first line of defense against HIV. The functionality of these cells in infants is often limited, underscoring the need for a deeper understanding of innate immune responses and their role in HIV acquisition. The adaptive immune response, involving T and B cells, is crucial for controlling viral infections. The maturation and functionality of these immune cells in infants significantly influence their ability to respond to HIV exposure.

## 1. Introduction

Human immunodeficiency virus (HIV) acquisition in infants remains a significant global health challenge, particularly in regions where maternal HIV prevalence is high.^[1]^ The World Health Organization estimates that approximately 1.7 million children under the age of 15 are living with HIV, with most infections occurring through vertical transmission during pregnancy, childbirth, or breastfeeding. Infants possess an immune system that is still developing, making them particularly susceptible to infections, including HIV.^[2]^ The initial immune response to pathogens in infants differs significantly from that of older children and adults due to the immaturity of both the innate and adaptive immune systems. This immaturity can lead to inadequate immune responses, increasing the risk of HIV acquisition. In this context, exploring the various immunological factors that influence HIV susceptibility in infants is essential for understanding the mechanisms of vertical transmission and identifying potential intervention points.^[3,4]^ One of the key factors influencing HIV acquisition in infants is the role of maternal antibodies. Immunoglobulin G (IgG) is transferred from the mother to the fetus through the placenta, providing passive immunity that protects the infant from infections during the early months of life.^[5]^ However, while maternal antibodies can offer protection against various pathogens, they can also inhibit the infant’s ability to mount an effective immune response to HIV. This dual role of maternal antibodies highlights the complexity of immune interactions in HIV-exposed infants and underscores the need for a nuanced understanding of their impact on susceptibility to infection.^[6–9]^

Innate immune responses represent the first line of defense against viral infections. In infants, the innate immune system, which includes natural killer (NK) cells, macrophages, and dendritic cells, plays a crucial role in recognizing and responding to HIV.^[10]^ However, the functionality of these innate immune cells is often compromised in infants, potentially affecting their ability to control HIV acquisition. Investigating the maturation of innate immune responses in infants and their implications for HIV susceptibility is vital for identifying potential therapeutic targets and intervention strategies. Mucosal immunity is another critical factor influencing HIV acquisition, as mucosal surfaces are primary entry points for the virus during transmission.^[11,12]^ In infants, mucosal immunity is still developing, and factors such as the presence of maternal antibodies in mucosal secretions can impact susceptibility to HIV. Understanding the role of mucosal immunity, including the presence of secretory immunoglobulin A (sIgA) and local immune responses in the gastrointestinal tract, is essential for elucidating the mechanisms underlying vertical transmission and the factors that modulate HIV susceptibility in infants. The adaptive immune response, particularly the roles of T and B cells, is also pivotal in determining susceptibility to HIV infection. The development and functionality of these immune cells in infants influence their ability to mount effective responses to HIV exposure. CD4+ T helper cells are essential for coordinating the immune response, while CD8+ cytotoxic T lymphocytes play a critical role in eliminating infected cells.^[11,12]^ Understanding the dynamics of T and B cell development in infants and their impact on HIV acquisition is crucial for identifying potential strategies to enhance immune protection.

Cytokine signaling and the immune regulatory environment further influence the susceptibility of infants to HIV. The cytokine milieu in HIV-exposed infants can affect T and B cell responses, shaping overall immune responsiveness. Pro-inflammatory and anti-inflammatory cytokines play a critical role in regulating immune responses, and imbalances in these cytokines may contribute to increased susceptibility to HIV.^[13]^ Investigating the cytokine profiles in infants at risk of HIV acquisition can provide valuable insights into the immunological mechanisms that underlie their vulnerability. Genetic factors also play a significant role in shaping the immune responses of infants to HIV. Variations in immune-related genes may influence susceptibility to HIV acquisition, leading to variability in infection rates among infants.^[10,14]^ Understanding the genetic determinants of immune responses in this population can inform personalized prevention strategies and highlight potential targets for therapeutic interventions.

### 1.1. Aim

The aim of this review is to critically examine the immunological factors that influence HIV acquisition in infants, with a particular focus on how innate and adaptive immune responses, maternal antibodies, mucosal immunity, cytokine environments, genetic predispositions, and placental immunology contribute to either susceptibility or resistance to HIV transmission during the perinatal and breastfeeding periods.

## 2. Review methods

This narrative review was conducted to synthesize and analyze current knowledge on the immunological factors influencing HIV acquisition in infants. A systematic search of the literature was carried out using major biomedical databases, including PubMed, Google Scholar, Scopus, and Web of Science, focusing on articles published between 2000 and 2024. Keywords and search terms included combinations of “HIV transmission in infants,” “vertical transmission,” “neonatal immunity,” “maternal antibodies,” “placental immunity,” “cytokines,” “mucosal immunity,” “genetic susceptibility,” and “immune responses in HIV-exposed infants.” Both primary research articles and relevant reviews were considered. Inclusion criteria comprised peer-reviewed studies focusing on immunological mechanisms involved in mother-to-child transmission of HIV, immunological profiles of HIV-exposed but uninfected infants, and experimental studies on immune pathways affecting HIV susceptibility in neonates. Articles that were not published in English, did not focus on human subjects, or lacked direct relevance to the topic were excluded. Selected articles were screened by reviewing titles and abstracts, followed by full-text examination to assess their relevance and scientific rigor. Data were extracted and organized into thematic areas, including innate and adaptive immunity, maternal antibody responses, mucosal barriers, cytokine profiles, genetic factors, and placental immunology. This approach enabled a comprehensive synthesis of evidence highlighting the multifaceted immunological landscape that influences HIV acquisition in early life. The final selection of literature aimed to provide a balanced perspective from both high-income and low-resource settings, considering the global context of HIV burden in infants. Through this method, the review seeks to present a coherent narrative that informs both scientific understanding and potential clinical strategies for reducing vertical HIV transmission.

### 2.1. Ethical approval

No ethical approval was obtained as this is a narrative review.

## 3. Maternal antibodies

Maternal antibodies play a crucial role in shaping the immune landscape of infants and can significantly influence their susceptibility to infections, including HIV. During pregnancy, IgG^[15]^ antibodies are transferred from the mother to the fetus through the placenta, providing passive immunity that protects the infant from a variety of pathogens during the early months of life. This transfer of antibodies is a vital mechanism through which mothers can provide immunological protection to their newborns, particularly in the context of infections prevalent in the environment. The presence of maternal antibodies can have both beneficial and detrimental effects on the infant’s immune responses. On one hand, maternal IgG antibodies can neutralize pathogens and prevent early infections, thereby contributing to the infant’s survival and health. For instance, maternal antibodies can provide protection against respiratory viruses and other infectious agents during the critical period of immune maturation in infancy. However, the presence of these antibodies can also complicate the infant’s ability to mount an effective immune response to HIV.^[16]^ Specifically, maternal antibodies may interfere with the infant’s adaptive immune responses, inhibiting the production of their own antibodies against HIV.^[11,17]^ In the case of HIV, maternal antibodies can have a dual role. While they may offer some degree of protection against the virus, they can also mask the infant’s immune responses, making it challenging for the infant’s immune system to recognize and respond to the infection. Additionally, the timing and quantity of maternal antibody transfer can vary significantly among individuals, leading to variability in the levels of passive immunity provided to different infants. This variability can impact the susceptibility of infants to HIV acquisition, particularly in the context of exposure to the virus through vertical transmission during childbirth or breastfeeding.^[3]^

The dynamics of maternal antibody transfer and their impact on infant immunity are further complicated by the presence of HIV itself. In HIV-infected mothers, the quality and functionality of maternal antibodies may be altered, potentially affecting the degree of protection afforded to the infant.^[3,5]^ Studies have shown that while maternal HIV-specific antibodies can be transferred to the infant, their neutralizing capacity may be limited compared to those from uninfected mothers. This limitation raises concerns about the effectiveness of maternal antibodies in providing robust protection against HIV infection in exposed infants.^[4]^ Moreover, the decline of maternal antibodies over time can also influence the infant’s susceptibility to HIV. As the passive immunity provided by maternal antibodies wanes, the infant’s own immune system must take over in mounting responses to infections. If the infant’s immune system is not adequately developed or if it encounters HIV during this transitional period, the risk of HIV acquisition may increase. This underscores the importance of understanding the timing and duration of maternal antibody protection in the context of HIV transmission.^[5,18]^

## 4. Innate immune responses

The innate immune system serves as the first line of defense against pathogens, including HIV, and plays a crucial role in determining the susceptibility of infants to infections.^[16]^ In infants, the innate immune response is characterized by a range of cellular and molecular components that work together to detect and respond to viral infections. However, the functionality of these innate immune cells in infants is often limited compared to that of older children and adults, which can impact their ability to effectively control HIV acquisition. Key components of the innate immune response include NK cells, macrophages, dendritic cells, and various pattern recognition receptors (PRRs).^[10,14]^ NK cells are critical for the early detection and elimination of HIV-infected cells. They can directly kill infected cells and produce cytokines that help shape the adaptive immune response. In infants, the development and functionality of NK cells may be impaired, leading to reduced antiviral activity. Studies have shown that infant NK cells exhibit lower cytotoxic activity and cytokine production compared to those in adults, which could compromise the infant’s ability to control HIV during initial exposure. Macrophages and dendritic cells also play essential roles in the innate immune response to HIV. These antigen-presenting cells are responsible for recognizing and engulfing pathogens, as well as processing and presenting viral antigens to T cells.^[11]^ In infants, the maturation of macrophages and dendritic cells is a dynamic process that influences the efficiency of antigen presentation and the subsequent activation of adaptive immune responses. However, research indicates that the function of these antigen-presenting cells can be suboptimal in infants, which may affect their ability to mount effective immune responses against HIV.

The role of PRRs, such as toll-like receptors (TLRs), is vital in detecting viral components and initiating innate immune responses. PRRs recognize specific pathogen-associated molecular patterns and trigger signaling pathways that lead to the production of pro-inflammatory cytokines and interferons. In infants, the expression and functionality of TLRs can differ from that of adults, influencing the ability to detect and respond to HIV. This altered responsiveness of PRRs may contribute to the impaired ability of infants to mount effective innate immune responses against HIV.^[10]^ The presence of maternal antibodies can also modulate innate immune responses in infants. Maternal antibodies can interact with innate immune cells, influencing their activation and function.^[10,12,19]^ For example, the binding of maternal IgG to fragment crystallizable region (of antibody) receptors on NK cells and macrophages can enhance their activity, potentially providing some level of protection against infections. However, the impact of maternal antibodies on the innate immune responses to HIV remains complex, as they can also interfere with the ability of these cells to respond appropriately to viral exposure. Furthermore, the inflammatory environment in HIV-exposed infants can affect the innate immune responses.^[5,18]^ Chronic inflammation is often associated with HIV infection and can lead to immune dysregulation. In infants, this dysregulated immune environment may impair the functionality of innate immune cells and contribute to increased susceptibility to HIV acquisition. Understanding the interactions between innate immune responses and the inflammatory milieu in HIV-exposed infants is critical for identifying potential therapeutic targets.

## 5. Mucosal immunity

Mucosal immunity plays a critical role in protecting infants from infections, including HIV, particularly since mucosal surfaces are primary entry points for the virus during transmission. In infants, mucosal immunity is still developing, which can significantly influence their susceptibility to HIV acquisition. The mucosal surfaces, including the gastrointestinal tract, respiratory tract, and urogenital tract, are lined with specialized immune cells and secretions that form the first barrier against pathogens. sIgA^[7,15]^ is a key component of mucosal immunity and plays a vital role in neutralizing viruses and preventing their entry into the body. In infants, sIgA is primarily derived from maternal colostrum and breast milk, providing passive immunity during the early months of life. The presence of maternal sIgA in breast milk can help protect infants from various infections; however, its role in providing specific protection against HIV is complex and not fully understood. Mucosal immune responses in infants are influenced by several factors, including the presence of maternal antibodies and the infant’s own immune development. Maternal antibodies, particularly sIgA and IgG, can be transferred through breastfeeding, offering some degree of protection against HIV. However, the transfer of maternal antibodies can also impact the infant’s ability to develop their own immune responses. The presence of maternal antibodies in the infant’s mucosal tissues may interfere with the recognition of HIV, potentially increasing susceptibility to infection during breastfeeding.

In addition to antibodies, various immune cells reside in mucosal tissues and contribute to the immune response against HIV. Dendritic cells, for instance, play a crucial role in capturing HIV at mucosal surfaces and presenting viral antigens to T cells. The maturation and function of these dendritic cells in infants are vital for establishing effective immune responses. However, studies have shown that dendritic cells in infants may have reduced functionality, which can compromise their ability to initiate appropriate immune responses to HIV. The cytokine environment at mucosal sites also influences immune responses to HIV.^[20]^ Cytokines are signaling molecules that modulate immune responses, and their balance can significantly impact susceptibility to infections. In HIV-exposed infants, the presence of specific cytokines can either enhance or inhibit mucosal immune responses. For example, pro-inflammatory cytokines may promote immune activation, while anti-inflammatory cytokines may dampen responses. Moreover, the role of gut microbiota in shaping mucosal immunity cannot be overlooked. The gut microbiota plays a crucial role in the development of the immune system, and alterations in the composition of gut bacteria may impact immune responses in infants. Studies have suggested that dysbiosis, or an imbalance in gut microbiota, can influence the susceptibility of infants to infections, including HIV. Investigating the interactions between gut microbiota, mucosal immunity, and HIV acquisition is an emerging area of research that may provide new insights into preventive strategies.^[5]^

## 6. Role of T cells

T cells play a pivotal role in the adaptive immune response against infections, including HIV. These immune cells are essential for recognizing and eliminating infected cells, orchestrating immune responses, and providing long-term protection through memory formation. In infants, the development and functionality of T cells significantly influence their susceptibility to HIV acquisition, making it crucial to understand the dynamics of T cell responses in this population. T cells can be categorized into 2 main subsets: CD4+ T helper cells and CD8+ cytotoxic T lymphocytes (CTLs). CD4+ T cells are essential for coordinating immune responses by helping activate other immune cells, including B cells and CTLs. In the context of HIV, CD4+ T cells are particularly vulnerable, as the virus specifically targets and infects these cells. The depletion of CD4+ T cells is a hallmark of HIV infection and is directly linked to the progression of the disease. In infants, the presence of sufficient CD4+ T cells is vital for maintaining immune competence and effectively responding to HIV exposure.^[11]^ In contrast, CD8+ CTLs are responsible for directly killing HIV-infected cells and producing cytokines that help control viral replication. The development and activation of CTLs are crucial for limiting the spread of HIV within the host. However, the functionality of CD8+ T cells in infants may differ from that of older children and adults. Infants often exhibit a reduced ability to mount robust CTL responses, which can compromise their ability to control HIV infection. Understanding the maturation of CTLs in infants and the factors that influence their activity is essential for identifying potential therapeutic strategies to enhance immune protection.^[9]^

The cytokine environment also plays a significant role in shaping T-cell responses to HIV. Cytokines, such as interleukin (IL)-2 and interferon-gamma, are crucial for T cell activation, proliferation, and differentiation. In HIV-exposed infants, the balance of pro-inflammatory and anti-inflammatory cytokines can influence the development and functionality of T-cell responses.^[12]^ For instance, an inflammatory cytokine milieu may enhance T cell activation, while excessive inflammation may lead to T cell exhaustion, characterized by reduced functionality and persistence. Another critical aspect to consider is the impact of maternal HIV infection on the infant’s T-cell responses. Infants born to HIV-infected mothers may be exposed to maternal HIV-specific antibodies that can modulate their immune responses. While these antibodies can offer some protection, they may also interfere with the infant’s ability to develop effective T-cell responses against HIV.^[21]^ The interplay between maternal antibodies and T cell development in infants is an area that warrants further investigation, as it may have implications for HIV susceptibility and the effectiveness of potential therapeutic interventions. Moreover, the phenomenon of T cell memory is crucial for long-term protection against HIV. Memory T cells, which arise following an initial immune response, can provide rapid and robust responses upon reexposure to the same pathogen. In infants, the generation of effective T cell memory is essential for establishing durable protection against HIV.^[22]^ However, factors such as the timing of HIV exposure, the presence of maternal antibodies, and the overall maturation of the immune system can influence the development and maintenance of memory T cells in infants.^[23]^

## 7. Role of B cells and antibody production

B cells are critical components of the adaptive immune system, primarily responsible for the production of antibodies that recognize and neutralize pathogens, including HIV.^[24]^ The role of B cells in the immune response is particularly important in infants, who face unique challenges in developing effective antibody responses due to the immaturity of their immune systems. B cell development begins in the bone marrow, where precursor cells undergo a series of maturation steps to become functional B cells. This process involves the rearrangement of immunoglobulin genes, allowing B cells to produce unique antibodies capable of recognizing specific antigens. Once mature, B cells migrate to peripheral lymphoid organs, such as the spleen and lymph nodes, where they can encounter antigens and initiate immune responses.^[11]^ In infants, the maturation of B cells is influenced by several factors, including maternal antibodies, cytokines, and the overall state of the immune system. Studies have shown that B cell development in infants may be delayed or impaired compared to older children and adults, which can impact their ability to mount effective antibody responses to infections.^[20]^ Upon encountering an antigen, B cells undergo activation, proliferation, and differentiation into antibody-secreting plasma cells or memory B cells. Plasma cells are responsible for producing large quantities of antibodies, while memory B cells provide long-term immunity by rapidly responding to subsequent exposures to the same pathogen. In the context of HIV, the ability of B cells to generate effective antibody responses is critical for controlling viral replication and preventing infection.^[25]^ However, infants often exhibit limited antibody production, which may contribute to their increased susceptibility to HIV acquisition.

The presence of maternal antibodies, particularly IgG, can significantly influence B cell responses in infants. Maternal antibodies provide passive immunity during the early months of life, protecting infants from various infections. However, these antibodies can also inhibit the infant’s B cell responses by masking viral antigens and preventing the recognition of HIV.^[8]^ This phenomenon, known as antibody-mediated immune suppression, can hinder the development of effective antibody responses against HIV in infants. Additionally, the dynamics of maternal antibody transfer and the timing of their decay are critical factors that can impact the infant’s susceptibility to HIV. The quality of the antibody response generated by B cells is also a crucial consideration. Effective neutralizing antibodies are essential for controlling HIV infection, and the ability of B cells to produce such antibodies can be influenced by various factors, including the presence of cytokines and the type of antigens encountered. In infants, the maturation of B cell responses and the generation of high-affinity, neutralizing antibodies may be impaired compared to adults. In addition to traditional B cell functions, recent studies have highlighted the importance of B cell memory and its role in long-term immunity against infections. Memory B cells can persist for years after an initial infection or vaccination, providing rapid and robust antibody responses upon reexposure. In infants, the development of memory B cells is critical for establishing durable protection against HIV. However, factors such as maternal antibodies, the timing of HIV exposure, and the overall state of the infant’s immune system can influence the generation and maintenance of B cell memory.^[11]^ Furthermore, the interplay between B cells and T cells is vital for orchestrating effective immune responses. Helper T cells (CD4+) provide essential signals for B cell activation and differentiation, while B cells can also present antigens to T cells, enhancing the overall immune response. In infants, the maturation of T cell responses and their interaction with B cells can significantly impact antibody production and the overall efficacy of the immune response to HIV.

## 8. Cytokine signaling and immune regulation

Cytokines are critical signaling molecules that mediate and regulate immune responses, playing a pivotal role in shaping the outcome of infections, including HIV. These small proteins, produced by various immune cells, facilitate communication between cells and orchestrate the immune response by influencing the activation, proliferation, and differentiation of T cells, B cells, and other immune components.^[6]^ In infants, the cytokine milieu is particularly important, as it can significantly impact their susceptibility to HIV acquisition and the overall effectiveness of their immune responses. The balance of pro-inflammatory and anti-inflammatory cytokines is crucial for maintaining immune homeostasis. Pro-inflammatory cytokines, such as IL-1, IL-6, and tumor necrosis factor-alpha, promote immune activation and facilitate the clearance of pathogens. In contrast, anti-inflammatory cytokines, such as IL-10 and transforming growth factor-beta (TGF-β), help regulate immune responses and prevent excessive inflammation.^[15]^ In infants, the immature immune system can lead to an imbalance in cytokine production, potentially resulting in inadequate or dysregulated immune responses to HIV. The role of cytokines in HIV infection is multifaceted. Certain cytokines can enhance the recruitment and activation of immune cells, facilitating the recognition and elimination of HIV-infected cells. For example, cytokines such as interferon-gamma are crucial for activating macrophages and enhancing the cytotoxic activity of CD8+ T cells.^[12]^ However, the cytokine response can also be shaped by the presence of HIV itself. The virus can induce a pro-inflammatory environment that may lead to immune activation and dysfunction, which is often observed in chronic HIV infection. In infants, the consequences of such cytokine dysregulation can be particularly pronounced, as their developing immune systems may struggle to cope with the effects of chronic inflammation.^[19]^

Moreover, the cytokine environment in HIV-exposed infants can influence the maturation of T cells and B cells. For instance, the presence of specific cytokines can drive the differentiation of naïve T cells into effector T cells or memory T cells, shaping the adaptive immune response. Similarly, cytokines such as IL-4 and IL-5 can promote B cell activation and antibody production.^[5]^ In infants, the maturation of these immune responses is critical for establishing effective protection against HIV, and any disruption in cytokine signaling may hinder their ability to respond to viral exposure. In addition to their roles in promoting immune activation, cytokines are also involved in immune regulation. Regulatory T cells (Tregs), which produce cytokines such as IL-10 and TGF-β, play a vital role in maintaining immune tolerance and preventing excessive immune activation. In the context of HIV infection, the presence of Tregs can be beneficial by controlling inflammation; however, an overactive Treg response may suppress effective immune responses against the virus, potentially increasing susceptibility to infection.^[1,2,26]^ The influence of maternal HIV infection on cytokine signaling in infants is another important consideration. Infants born to HIV-positive mothers may be exposed to altered cytokine profiles during pregnancy and breastfeeding. Maternal HIV infection can lead to the production of cytokines that may influence the infant’s immune development and responses. Additionally, the presence of maternal antibodies can also modulate cytokine signaling in infants, affecting their overall immune responses to infections, including HIV.^[10]^

## 9. Genetic factors

Genetic factors play a significant role in determining the susceptibility of infants to HIV acquisition, influencing various aspects of the immune response, viral transmission, and disease progression.^[18]^ The interplay between host genetics and the immune system can modulate the ability to control viral infections, including HIV, and understanding these genetic factors is crucial for elucidating the risk of infection in HIV-exposed infants. One of the key genetic factors influencing susceptibility to HIV is the variation in genes associated with the immune response. For instance, polymorphisms in human leukocyte antigen (HLA) genes can significantly impact the effectiveness of T cell responses against HIV.^[24]^ HLA molecules are essential for presenting viral peptides to T cells, and certain HLA types are associated with better control of HIV infection. Infants with HLA alleles that enhance the recognition of HIV-infected cells may have a reduced risk of acquiring the virus compared to those with less effective HLA types.^[21]^ Additionally, variations in other immune-related genes, such as cytokine genes, can influence the production and signaling of cytokines that modulate immune responses, thereby affecting susceptibility to HIV. In addition to HLA genes, the presence of certain co-inhibitory receptors on T cells can impact the immune response to HIV. For example, programmed cell death protein-1 is a co-inhibitory receptor that, when upregulated, can lead to T cell exhaustion and diminished antiviral activity. Genetic variations that influence the expression or function of programmed cell death protein-1 may affect the capacity of T cells to control HIV infection.^[11]^

Genetic factors also extend to the influence of innate immune responses on HIV susceptibility. Variants in genes associated with PRRs can affect the ability of innate immune cells to detect and respond to HIV.^[14]^ For instance, polymorphisms in TLR genes may modulate the activation of innate immune pathways and influence the initial immune response to HIV exposure. Infants with genetic variants that enhance PRR signaling may have an improved capacity to respond to HIV and reduce the likelihood of acquiring the virus. Moreover, genetic factors can influence the efficiency of maternal antibody transfer and the subsequent immune responses in infants.^[9]^ Genetic polymorphisms in fragment crystallizable region (of antibody) receptor genes may affect the binding and signaling of maternal antibodies, potentially modulating their ability to provide passive immunity. Infants with favorable genetic profiles that enhance the activity of maternal antibodies may have a lower risk of HIV acquisition compared to those with less effective antibody responses. In addition to specific genetic polymorphisms, the overall genetic background of an individual can also play a role in immune responses to HIV.^[27]^ Genetic diversity within populations can result in varying susceptibility to infections and differing immune responses. Studies have shown that populations with specific genetic traits may have different rates of HIV transmission and progression. Understanding the genetic diversity in HIV-exposed infants and its implications for susceptibility is an important area of research. Finally, it is important to consider the influence of epigenetic factors, which can modulate gene expression without altering the underlying DNA sequence.^[28]^ Epigenetic modifications can be influenced by environmental factors, including maternal health and nutrition, and can have lasting effects on the developing immune system. These modifications may impact the expression of genes involved in immune responses, potentially affecting the infant’s susceptibility to HIV acquisition.^[2,7,18]^

## 10. Conclusion

The susceptibility of infants to HIV acquisition is a multifaceted issue influenced by a complex interplay of immunological factors. As the first line of defense, the innate immune system, including components such as natural killer cells and macrophages, plays a critical role in early recognition and response to HIV. However, the functional limitations of these innate immune cells in infants can compromise their ability to control viral exposure effectively. Mucosal immunity is another crucial aspect of the infant immune response, as the mucosal surfaces are primary entry points for HIV. The development of robust mucosal immune responses, including the production of sIgA and the activation of dendritic cells, is essential for preventing infection. Additionally, T cells and B cells are vital for adaptive immunity, with their development and functionality being critical determinants of susceptibility. The interactions between these immune cells, along with the influence of cytokines, shape the overall immune response to HIV in infants.

## Author contributions

**Conceptualization:** Emmanuel Ifeanyi Obeagu.

**Methodology:** Emmanuel Ifeanyi Obeagu, Isaac Isiko, Getrude Uzoma Obeagu.

**Resources:** Emmanuel Ifeanyi Obeagu.

**Supervision:** Emmanuel Ifeanyi Obeagu.

**Validation:** Emmanuel Ifeanyi Obeagu, Isaac Isiko, Getrude Uzoma Obeagu.

**Visualization:** Emmanuel Ifeanyi Obeagu, Getrude Uzoma Obeagu.

**Writing – original draft:** Emmanuel Ifeanyi Obeagu, Isaac Isiko, Getrude Uzoma Obeagu.

**Writing – review & editing:** Emmanuel Ifeanyi Obeagu, Isaac Isiko, Getrude Uzoma Obeagu.
